# Association of physical activity and sedentary behavior with stages of cardiovascular–kidney–metabolic syndrome among U.S. adults: NHANES 2007–2020^☆^

**DOI:** 10.1016/j.ahjo.2025.100639

**Published:** 2025-10-14

**Authors:** Shuo Pang, Yuxi Dongye, Yingwei Bi, Jianbo Wang

**Affiliations:** a Department of Urology, The First Affiliated Hospital of Dalian Medical University, Dalian, 116011, China; b Department of Breast Surgery, Jinan Third People's Hospital, Jinan, Shandong, 250101, China

**Keywords:** Cardiovascular–kidney–metabolic, Moderate-to-vigorous physical activity, Sedentary behavior, NHANES

## Abstract

**Background:**

Cardiovascular–kidney–metabolic (CKM) syndrome, newly defined by the American Heart Association, integrates metabolic risk factors, chronic kidney disease, and cardiovascular dysfunction. Although clinically important, evidence-based prevention strategies remain limited. This study examined associations of moderate-to-vigorous physical activity (MVPA) and sedentary behavior (SB) with CKM stage in a representative U.S. adult sample.

**Methods:**

We used survey-weighted multinomial logistic regression and restricted cubic splines (RCS). MVPA was classified as 0, 1–149, and ≥ 150 min/week; SB as <5, 5–8, and ≥ 8 h/day; CKM was staged 0–4 (stage 0 as reference). Predefined subgroups were age (20–59 vs 60–79 years), sex (female vs male) and PIR (high >3.49; medium >1.49–<3.49; low ≤1.49).

**Results:**

Compared with individuals reporting no MVPA, those achieving ≥150 min/week had approximately 33 % lower odds of CKM stage 1 (OR 0.67, 95 % CI 0.46–0.97), 44 % lower odds of stage 2 (OR 0.56, 95 % CI 0.38–0.83), 92 % lower odds of stage 3 (OR 0.09, 95 % CI 0.02–0.32), and 65 % lower odds of stage 4 (OR 0.35, 95 % CI 0.19–0.63). Subgroup analyses by age, sex, poverty–income ratio, and SB indicated that the protective impact of MVPA was evident across age and socioeconomic subgroups, with relatively stronger associations among younger adults and benefits at both low and high socioeconomic strata. By contrast, SB showed weaker independent associations with CKM severity after full adjustment.

**Conclusions:**

Greater MVPA was associated with lower odds of advanced CKM stage. These findings highlight the role of physical activity in CKM prevention and support public health strategies to reduce lifestyle-related risks.

## 1 Introduction

Cardiovascular diseases frequently co-occur with renal disorders and metabolic conditions such as obesity and type 2 diabetes. Growing evidence on the underlying pathophysiology has drawn attention to the intricate links among metabolic risk factors, chronic kidney disease (CKD), and cardiovascular health [1]. Cardiovascular-kidney-metabolic (CKM) syndrome, as defined by the American Heart Association, integrates interconnected risks of obesity, metabolic dysfunction, chronic kidney disease, and cardiovascular disease, with staging from 0 to 4 [1]. In the United States, more than one in four adults exhibits at least one of these conditions, underscoring the clinical importance of this multimorbidity [2]. In October 2023, the American Heart Association published a scientific statement highlighting these interrelationships, designating the overlapping negative effects of these conditions as CKM syndrome [1].

Beyond lifestyle, CKM susceptibility reflects adiposity and insulin resistance, hypertension and dyslipidemia, and kidney function—as emphasized by recent statements and position papers—highlighting the need to evaluate modifiable behaviors alongside these core drivers [3]. Physical activity (PA) levels and sedentary behavior (SB) are potentially modifiable factors that influence health outcomes [4,5]. Numerous studies have shown that regular exercise confers substantial benefits on inflammation, metabolic processes, cardiac function, and renal performance in various populations [6]. In contrast, prolonged SB has been associated with heightened risks of type 2 diabetes mellitus (T2DM), coronary artery disease (CAD), heart failure, cardiovascular events, chronic kidney disease, and increased all-cause mortality [7,8]. Only a few studies have investigated the relationship between CKM and physical activity. For example, Wang et al. reported a nonlinear association between PA and early CKM risk [9].

Elucidating the association between different CKM stage and physical activity levels is crucial for refining risk assessment, informing early preventive strategies, and improving long-term patient outcomes. Therefore, this study aimed to examine the link between physical activity and the distinct stages of CKM syndrome among U.S. adults, utilizing data from the National Health and Nutrition Examination Survey (NHANES).

## 2 Materials and methods

### 2.1 Study population

We conducted a cross-sectional analysis to examine the association between physical activity (PA) and various stages of CKM syndrome in U.S. adults, using data from the National Health and Nutrition Examination Survey (NHANES) conducted between 2007 and 2020 (available at: https://www.cdc.gov/nchs/nhanes/index.htm). NHANES, administered by the Centers for Disease Control and Prevention (CDC) through the National Center for Health Statistics (NCHS), is a nationally representative program that integrates interviews, physical examinations, laboratory tests, and dietary assessments. The study protocol was approved by the NCHS Research Ethics Review Board, and written informed consent was obtained from all participants or their legal guardians.

**Fig. 1:**
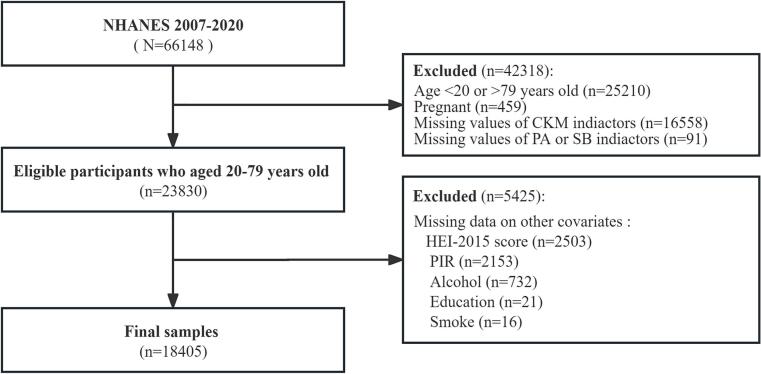
Flowchart of participants included in analyses. **Abbreviations:** CKM: cardiovascular-kidney-metabolic; HEI-2015: Healthy Eating Index-2015; SB: sedentary behavior; PIR: poverty–income ratio; PA: physical activity.

Study population. We pooled data from NHANES 2007–2020 (*n* = 66,148). We sequentially excluded pregnant participants (*n* = 459), those outside the 20–79-year age range (*n* = 25,210), participants with insufficient information to assign a CKM stage (*n* = 16,558), and those missing physical activity (PA) or sedentary behavior (SB) data (*n* = 91), leaving 23,830 nonpregnant adults aged 20–79 years with CKM staging and PA/SB data. Among these, we further excluded participants with missing key covariates (HEI-2015 score, PIR, alcohol, education, or smoking) (*n* = 5425), resulting in a final analytic sample of 18,405. The selection flow is shown in Fig. 1. While this complete-case approach is standard in NHANES analyses, it may introduce selection bias if excluded individuals differ systematically from those retained (e.g., in socioeconomic or health profiles). We acknowledge this as a limitation in the Discussion. (Fig. 1).

### 2.2 Assessments of CKM syndrome

**Fig. 2:**
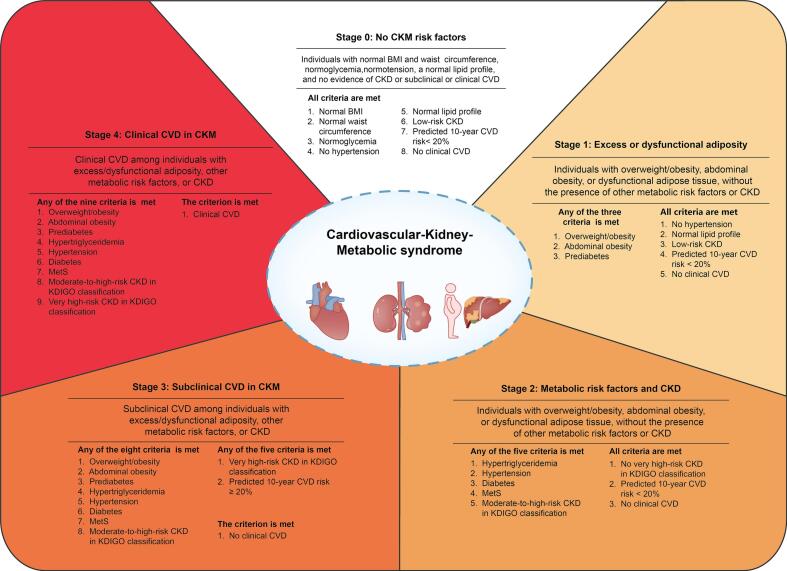
Methods for evaluating each CKM stage. **Abbreviations:** BMI: body mass index; MVPA: moderate-to-vigorous physical activity; CKD: chronic kidney disease; CKM: cardiovascular-kidney-metabolic; CVD: cardiovascular disease; DBP: diastolic blood pressure; eGFR: estimated glomerular filtration rate; HDL: high-density lipoprotein; KDIGO: The Kidney Disease: Improving Global Outcomes; SBP: systolic blood pressure; UACR: urinary albumin to creatinine ratio. Definition: Normal BMI: BMI <25 kg/m^2^ (or < 23 kg/m^2^ if Asian ancestry); normal waist circumference: waist circumference < 88/102 cm in female/male (or if Asian ancestry <80/90 cm in female/male); normoglycemia: fasting blood glucose <100 mg/dl and HbA1c < 5.7 % and without self-reported diagnosis of diabetes, use of insulin, or oral hypoglycemic agents; no hypertension: SBP <130 mmHg and DBP <80 mmHg without self-reported diagnosis of hypertension or use of antihypertensive medications; normal lipid profile: HDL cholesterol ≥50/40 mg/dl in female/male and triglycerides <150 mg/dl; low-risk CKD: Low-risk CKD in KDIGO classification according to eGFR and UACR: UACR <30 mg/g and eGFR ≥60 ml/min/1.73m^2^.* Asian was not listed as a separate race/ethnicity until NAHNES 2011–2012, therefore the uniform threshold for BMI and waist circumference was used in all participants in NHANES 2007–2010. Note: Because NHANES did not separately identify Asian race/ethnicity in 2007–2010, uniform BMI and waist thresholds were applied across all cycles in this study.

Operationalization in NHANES. We operationalized AHA CKM stage using NHANES variables. Clinical CVD (stage 4) included any prior heart failure, coronary heart disease, myocardial infarction, or stroke. Subclinical CVD (stage 3) was assigned if the predicted 10-year CVD risk ≥20 % (simplified CKM risk algorithm; components in Table S2) [10] or if high-risk CKD per KDIGO was present. CKD risk categories followed KDIGO using eGFR and UACR thresholds (Table S1). Stage 0–2 were determined from adiposity and metabolic indicators (Table S1). [3,11] Mapping of variables to stages is illustrated in Fig. 2.

### 2.3 Assessments of physical activity and sedentary behavior

**Fig. 3:**
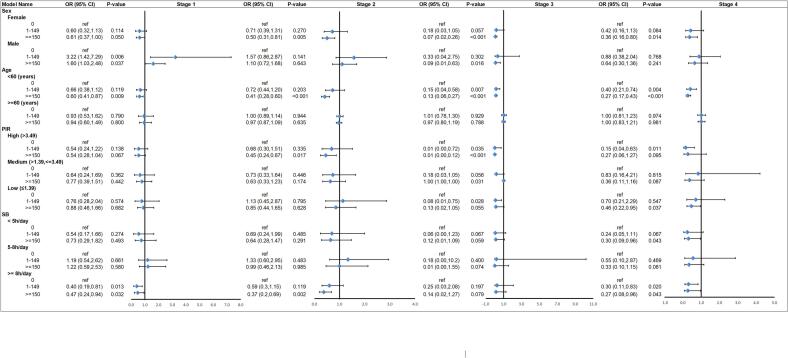
The relationship between MVPA and each CKM stage in different subgroups. **Abbreviations:** SB: sedentary behavior; MVPA: moderate-to-vigorous physical activity; PIR: poverty–income ratio. Results are expressed as multivariable-adjusted odds ratio after controlling covariates including age, sex, race/ethnicity, Healthy Eating Index-2015, educational level (above high school, high school or equivalent, under high school), marital status (married/cohabiting, never married, widowed/divorced/separated), tobacco use (current, former, and never), alcohol use (heavy, mild, moderate, and never), PIR [high (>3.49), low (≤1.49), medium (>1.49, < 3.49)], and sedentary behavior(<5 h/day, 5–8 h/day, or ≥ 8 h/day).

The National Health and Nutrition Examination Survey (NHANES) Physical Activity Questionnaire (PAQ), developed by the World Health Organization (WHO), was used to collect information on PA and SB from 2007 to 2020. Trained interviewers administered the PAQ in participants' homes using the Computer-Assisted Personal Interview (CAPI) system. The NHANES dataset provides standard MET values linked to different exercise intensities, which serve as reference points for quantifying exertion levels. (See Fig. 3.)

PA was estimated using two complementary approaches. First, total weekly MET-minutes (MET × weekly frequency × duration) were calculated to capture the type, frequency, and duration of each reported activity [4]. Second, overall PA duration (min/week) was derived by combining moderate and vigorous activities, with one minute of vigorous activity considered equivalent to two minutes of moderate activity [4,12]. This method aligns with the American Physical Activity Guidelines, which recommend 150 min of moderate-intensity or 75 min of vigorous-intensity exercise per week, corresponding to approximately 600 MET-min/week [4]. In keeping with these guidelines, MVPA was classified into three levels: inactivity (0 min/week), partially active (1–149 min/week), and fully active (≥150 min/week) [4].

Sedentary behavior was defined as time spent sitting at school, at home, during transportation, or in leisure activities such as reading, watching television, and using a computer. The measurement of SB relied on self-reported hours per day typically spent in seated positions [13,14]. SB time was then categorized into three groups: <5 h/day, 5–8 h/day, or ≥ 8 h/day [15].

### 2.4 Assessments of other covariates

This study considered several demographic variables, including participants' age, sex, and race/ethnicity (non-Hispanic White, non-Hispanic Black, Mexican, other Hispanic, and other race/multiracial, including Asian). Additional self-reported lifestyle factors encompassed marital status (married/cohabiting, never married, widowed/divorced/separated), educational level (above high school, high school or equivalent, below high school), tobacco use (current, former, never), alcohol use (former, heavy, mild, moderate, never) [16], and the Income-to-Poverty Ratio (PIR) categorized as high (>3.49), medium (>1.49 to <3.49), or low (≤1.49) [17]. Dietary quality was assessed using the Healthy Eating Index–2015 (HEI–2015), which ranges from 0 to 100 and is derived from a 24-h dietary recall [18]. The HEI–2015 is a validated metric that captures overall adherence to recommended eating patterns among Americans.

### 2.5 Statistical analysis

Analyses were conducted in accordance with the National Center for Health Statistics (NCHS) guidelines for complex, multistage, probability sampling, which account for both stratification and primary sampling units. Continuous variables were described using means with standard deviation (SD), and categorical variables were presented as unweighted counts with weighted percentages. The Kruskal-Wallis test was applied to compare continuous variables across the five CKM stages (0–4). For categorical variables, Pearson's chi-square test with Rao–Scott adjustment was used to account for the complex survey design [ 19].

A survey-based multinomial logistic regression model was fitted to examine the associations between levels of moderate-to-vigorous physical activity (MVPA) and the four CKM stages, using stage 0 as the reference. Odds ratios (ORs) and 95 % confidence intervals (CIs) were obtained. For the crude model, no confounding factors were adjusted, and we fitted a partially adjusted model that included age, sex, and ethnicity. Finally, we fitted a fully adjusted model that included tobacco and alcohol users, HEI-2015 score, PIR, and SB. Restricted cubic spline (RCS) regression with knots at the 5th, 35th, 65th, and 95th percentiles was used to explore nonlinear relationships between physical activity and CKM. Additionally, a linear trend test was performed to evaluate the pattern of increasing or decreasing risk across the three MVPA categories (0, 1–149, ≥150 min/week) within each CKM stage. To improve robustness to self-report measurement error, we triangulated MVPA using two prespecified operationalizations—categorical minutes/week (0, 1–149, ≥150) and continuous MET-minutes/week with restricted cubic splines—and compared inferences across parameterizations. Because MVPA and SB may be behaviorally correlated, we included both in the same multinomial models and additionally examined SB-stratified associations.

We conducted supplementary analyses to assess the robustness of our findings. Subgroup analyses were stratified by age (20–59 vs. 60–79 years), sex (female vs. male), sedentary behavior (< 5 h/day, 5-8 h/day, ≥ 8 h/day) and PIR [high (>3.49), medium (>1.49 to <3.49), low (≤1.49)] to account for demographic variations. Subgroup analyses (age, sex, PIR, SB strata) were pre-specified and limited in number. We emphasized effect-size patterns with 95 % CIs and treated *p*-values as descriptive; no formal multiplicity adjustment was applied. Findings from subgroup analyses should therefore be interpreted as exploratory.

All analyses were conducted using R 4.3.2 (R Foundation for Statistical Computing, Vienna, Austria). Statistical significance was defined as a two-sided p-value <0.05.

## 3 Results

### 3.1 Baseline characteristics

Our study included 18,405 participants aged 20–79 years (mean 50.2 [15.5]); 9319 were men, and 7426 were non-Hispanic White. There were 11,918 participants at CKM stage 2. Regarding MVPA, 4891 reported no MVPA, 2623 reported 1–149 min/week, and 10,891 reported ≥150 min/week (Table 1). Compared with participants with high CKM stage, participants with lower CKM stage were younger, less likely to be current smokers or drinkers, more likely to have higher HEI-2015 scores, and more likely to spend more time on physical activity and spend less time sitting. (Table 1).

**Table 1:** Characteristics of Participants by CKM stage.

Overall		CKM					
Characteristic⁎	*N* = 18,405 (100 %)^1^	0, *N* = 547 (4.2 %)^1^	1, *N* = 2328 (15 %)^1^	2, *N* = 11,918 (62 %)^1^	3, *N* = 1197 (7.4 %)^1^	4, *N* = 2415 (11 %)^1^	P Value^2^
Age (years)⁎	50.2 (15.5)	33.6 (11.2)	41.1 (13.7)	49.3 (13.8)	69.8 (8.0)	62.0 (11.8)	**<0.001**
Sex⁎-Male	9319.0 (50.8 %)	197.0 (35.3 %)	1091.0 (48.7 %)	5837.0 (49.7 %)	768.0 (63.8 %)	1426.0 (57.0 %)	**<0.001**
Marital⁎							**<0.001**
Married/cohabiting	9346.0 (57.4 %)	263.0 (54.2 %)	1247.0 (59.2 %)	6027.0 (57.0 %)	671.0 (62.3 %)	1138.0 (54.7 %)	
Married/Living with Partner	1772.0 (7.8 %)	0.0 (0.0 %)	199.0 (6.3 %)	1246.0 (8.7 %)	96.0 (7.2 %)	231.0 (8.7 %)	
Never married	2856.0 (14.9 %)	237.0 (37.5 %)	560.0 (21.8 %)	1786.0 (14.1 %)	63.0 (4.3 %)	210.0 (7.8 %)	
Widowed/divorced/separated	4428.0 (19.9 %)	47.0 (8.3 %)	322.0 (12.7 %)	2856.0 (20.2 %)	367.0 (26.2 %)	836.0 (28.8 %)	
Race⁎, ‡							**<0.001**
Mexican American	2663.0 (7.9 %)	36.0 (4.5 %)	394.0 (10.1 %)	1970.0 (9.0 %)	30.0 (0.8 %)	233.0 (4.5 %)	
Non-Hispanic Black	4239.0 (10.9 %)	79.0 (7.0 %)	433.0 (9.2 %)	3019.0 (12.3 %)	58.0 (1.8 %)	650.0 (13.2 %)	
Non-Hispanic White	7426.0 (68.4 %)	253.0 (73.1 %)	891.0 (66.0 %)	4079.0 (64.8 %)	1073.0 (96.1 %)	1130.0 (71.4 %)	
Other Hispanic	1861.0 (5.3 %)	51.0 (6.0 %)	257.0 (6.4 %)	1320.0 (5.7 %)	16.0 (0.5 %)	217.0 (4.3 %)	
Other Race - Including Multi-Racial	2216.0 (7.5 %)	128.0 (9.4 %)	353.0 (8.3 %)	1530.0 (8.2 %)	20.0 (0.7 %)	185.0 (6.5 %)	
Education⁎							**<0.001**
Above high school	9837.0 (61.1 %)	407.0 (76.9 %)	1456.0 (67.4 %)	6312.0 (60.8 %)	611.0 (57.0 %)	1051.0 (50.3 %)	
High school or equivalent	4262.0 (24.1 %)	93.0 (16.5 %)	468.0 (21.6 %)	2729.0 (23.9 %)	339.0 (28.3 %)	633.0 (28.8 %)	
Under high school	4306.0 (14.8 %)	47.0 (6.5 %)	404.0 (11.0 %)	2877.0 (15.3 %)	247.0 (14.7 %)	731.0 (20.9 %)	
PIR⁎							**<0.001**
High (>3.49)	5718.0 (44.2 %)	212.0 (50.5 %)	841.0 (47.5 %)	3791.0 (45.4 %)	352.0 (40.8 %)	522.0 (31.9 %)	
Low (≤1.49)	6288.0 (22.8 %)	159.0 (22.1 %)	679.0 (19.1 %)	4089.0 (23.0 %)	323.0 (16.9 %)	1038.0 (30.8 %)	
Medium (>1.49, ≤3.49)	6399.0 (33.1 %)	176.0 (27.5 %)	808.0 (33.4 %)	4038.0 (31.6 %)	522.0 (42.3 %)	855.0 (37.3 %)	
Tobacco user⁎							**<0.001**
Current smoker	3817.0 (19.2 %)	96.0 (16.7 %)	394.0 (15.2 %)	2505.0 (19.7 %)	207.0 (18.4 %)	615.0 (24.1 %)	
Former smoker	4865.0 (27.8 %)	72.0 (14.2 %)	475.0 (24.7 %)	2900.0 (26.1 %)	515.0 (41.9 %)	903.0 (38.4 %)	
Never smoker	9723.0 (52.9 %)	379.0 (69.1 %)	1459.0 (60.1 %)	6513.0 (54.3 %)	475.0 (39.7 %)	897.0 (37.6 %)	
Alcohol user⁎							**<0.001**
former	2669.0 (12.3 %)	33.0 (5.3 %)	173.0 (6.7 %)	1588.0 (11.4 %)	267.0 (19.2 %)	608.0 (23.3 %)	
heavy	3652.0 (20.5 %)	125.0 (23.7 %)	550.0 (23.1 %)	2552.0 (22.1 %)	100.0 (8.6 %)	325.0 (14.3 %)	
mild	6708.0 (39.4 %)	201.0 (36.4 %)	854.0 (39.1 %)	4197.0 (38.2 %)	556.0 (50.2 %)	900.0 (40.5 %)	
moderate	2965.0 (18.2 %)	124.0 (25.7 %)	484.0 (22.8 %)	1960.0 (18.6 %)	109.0 (10.0 %)	288.0 (12.2 %)	
never	2411.0 (9.6 %)	64.0 (8.9 %)	267.0 (8.3 %)	1621.0 (9.7 %)	165.0 (12.0 %)	294.0 (9.7 %)	
Age group (years)⁎							**<0.001**
<60	11,259.0 (68.8 %)	547.0 (100.0 %)	2033.0 (88.4 %)	7800.0 (74.5 %)	112.0 (10.2 %)	767.0 (36.0 %)	
≥ 60	7146.0 (31.2 %)	0.0 (0.0 %)	295.0 (11.6 %)	4118.0 (25.5 %)	1085.0 (89.8 %)	1648.0 (64.0 %)	
MVPA group (min/week)⁎							**<0.001**
0	4891.0 (22.8 %)	68.0 (9.9 %)	395.0 (14.9 %)	3070.0 (22.1 %)	401.0 (31.4 %)	957.0 (37.0 %)	
1–149	2623.0 (13.8 %)	66.0 (10.7 %)	278.0 (11.3 %)	1750.0 (14.6 %)	175.0 (13.2 %)	354.0 (14.0 %)	
≥ 150	10,891.0 (63.5 %)	413.0 (79.4 %)	1655.0 (73.7 %)	7098.0 (63.3 %)	621.0 (55.4 %)	1104.0 (49.0 %)	
MET total (MET-min/week)⁎	3682.5 (6069.5)	4700.2 (6893.7)	4816.4 (6895.7)	3699.7 (6092.7)	2291.6 (3861.3)	2524.1 (5097.0)	**<0.001**
HEI-2015⁎	51 (14)	53 (15)	51 (14)	50 (14)	52 (13)	50 (14)	**0.001**
SB (min/day)⁎	401.0 (511.2)	396.8 (388.2)	366.1 (253.9)	400.9 (525.8)	401.1 (405.9)	453.1 (747.8)	**<0.001**
SB (hours/day)⁎							**0.005**
< 5 h/day	7469.0 (35.9 %)	188.0 (32.0 %)	1017.0 (39.2 %)	5025.0 (36.4 %)	428.0 (32.8 %)	811.0 (32.1 %)	
5-8 h/day	4867.0 (26.9 %)	166.0 (29.9 %)	590.0 (25.4 %)	3054.0 (26.5 %)	369.0 (31.4 %)	688.0 (27.7 %)	
≥ 8 h/day	6069.0 (37.1 %)	193.0 (38.1 %)	721.0 (35.3 %)	3839.0 (37.1 %)	400.0 (35.8 %)	916.0 (40.2 %)	

CKM: cardiovascular-kidney-metabolic; HEI: Healthy Eating Index; PIR: poverty income ratio; MVPA: moderate-to-vigorous physical activity; SB: sedentary behavior; MET: metabolic equivalent.

1 Mean (SD) for continuous; Counts (n) are unweighted; percentages are survey-weighted column percentages unless otherwise specified.

2 Design-based Kruskal Wallis test; Pearson's X^2: Rao & Scott adjustment

⁎ Weighted to be nationally representative.

‡ Including Asian and multiracial.

### 3.2 Association between physical activity (PA), sedentary behavior (SB) and CKM

Fully adjusted models (Table 2) indicated that participants achieving ≥150 min/week of MVPA had appreciable reductions in the odds of each CKM stage compared with those reporting no MVPA. Specifically, the odds of CKM stage 1 were about 33 % lower (OR 0.67 [95 % CI, 0.46–0.97]), stage 2 were 44 % lower (OR 0.56 [95 % CI, 0.38–0.83]), stage 3 were 91 % lower (OR 0.09 [95 % CI, 0.02–0.32]), and stage 4 were 65 % lower (OR 0.35 [95 % CI, 0.20–0.63]). Although 1–149 min/week of MVPA was most clearly protective at CKM stage 3 and 4, a general trend of decreasing odds was observed as weekly MVPA increased across all stages. Unadjusted and partially adjusted findings (Table S3) were broadly consistent with the fully adjusted results. Higher total MET-minutes of MVPA consistently showed significant inverse associations with advanced CKM stage (2–4) across all models, whereas no significant effect was noted for stage 1 (Table S4).

**Fig. 4:**
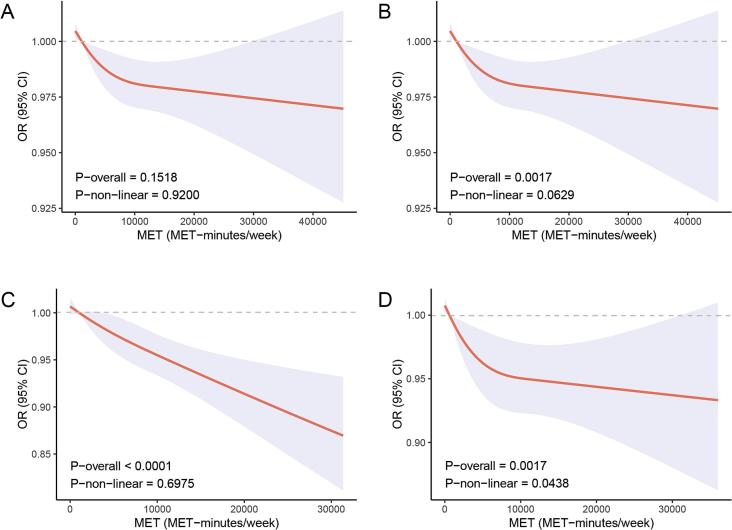
Restricted cubic splines (RCS) analysis investigating the relationship between MET-MVPA and each CKM stage. **Abbreviations:** RCS: Restricted cubic splines; PIR: poverty–income ratio; MET: metabolic equivalent; MVPA: moderate-to-vigorous physical activity. Adjustment include age, race/ethnicity, Healthy Eating Index-2015, educational level (above high school, high school or equivalent, under high school), marital status (married/cohabiting, never married, widowed/divorced/separated), tobacco use (current, former, and never), alcohol use (heavy, mild, moderate, and never), PIR [high (>3.49), low (≤1.49), medium (>1.49, < 3.49)], and sedentary behavior. The orange lines correspond to the central estimates, and the shaded regions indicate the 95 % confidence interval.

The restricted cubic spline (RCS) curves (Fig. 4) demonstrate the relationship between weekly MET-minutes of moderate-to-vigorous physical activity (MVPA) and each CKM stage. For CKM stage 1 (Panel A), no significant association was observed. In contrast, CKM stage (2–4) (Panels B—D) show a clear downward trend, indicating progressively lower odds of advanced CKM with increasing MVPA. CKM stage 3 exhibits a strongly linear decline, whereas CKM stage 4 displays both linear and mild nonlinear components. Collectively, these results highlight the protective role of higher MVPA, particularly in later CKM stage.

**Table 2:** Levels of MVPA level in Relation to CKM Stage 1 to 4 in Fully Adjusted Models.

	Stage 1				Stage 2				Stage 3				Stage 4			
Characteristic	OR^1^	95 % CI^1^	p-value	*p for trend*	OR^1^	95 % CI^1^	*p*-value	*p for trend*	OR^1^	95 % CI^1^	p-value	*p for trend*	OR^1^	95 % CI^1^	p-value	*p for trend*
MVPA group^†^ (min/week)				0.040				0.003				<0.001				<0.001
0	–	–			–	–			–	–			–	–		
1–149	0.61	0.36, 1.04	0.070		0.78	0.47, 1.30	0.333		0.14	0.03, 0.61	0.010		0.42	0.20, 0.86	0.018	
≥ 150	0.67	0.46, 0.97	0.036		0.56	0.38, 0.83	0.004		0.09	0.02, 0.32	<0.001		0.35	0.20, 0.63	<0.001	

**Abbreviations:** CI: confidence interval; CKM: cardiovascular-kidney-metabolic; OR: odds ratio; PIR: poverty income ratio; MVPA: moderate-to-vigorous physical activity.

Models were adjusted for age, race/ethnicity, Healthy Eating Index-2015, educational level (above high school, high school or equivalent, under high school), marital status (married/cohabiting, never married, widowed/divorced/separated), tobacco use (current, former, and never), alcohol use (heavy, mild, moderate, and never), PIR [high (>3.49), low (≤1.49), medium (>1.49, < 3.49)], sedentary behavior time, and was categorized into three groups (< 5 h/day, 5-8 h/day, and ≥ 8 h/day).

† MVPA was constructed by the summed time inactivity (0 min/week), low level of activity (1–149 min/week), and recommended activity level (≥ 150 min/week).

1 OR = Odds Ratio, CI = Confidence Interval

Subgroup analyses demonstrated that the protective associations of MVPA were not uniform across groups. By sex (Table S5), women achieving ≥150 min/week MVPA had significantly lower odds of CKM stage 2 (OR 0.50, 95 % CI 0.31–0.81), 3 (OR 0.07, 95 % CI 0.02–0.26), and 4 (OR 0.36, 95 % CI 0.16–0.80), whereas in men the strongest effect was observed for CKM stage 3 (OR 0.09, 95 % CI 0.01–0.63). By age (Table S6), marked benefits of MVPA ≥150 min/week were seen in adults <60 years for all stages (stage 2 OR 0.41, 95 % CI 0.28–0.60; stage 3 OR 0.13, 95 % CI 0.06–0.27; stage 4 OR 0.27, 95 % CI 0.17–0.43), while associations were null in those ≥60 years. By socioeconomic status (Table S7), benefits were evident in both high- and low-income groups, with strong inverse associations for stage 3 (high PIR OR 0.01, 95 % CI 0.00–0.12; low PIR OR 0.13, 95 % CI 0.02–1.05) and stage 4 (low PIR OR 0.46, 95 % CI 0.22–0.95). Collectively, these results suggest that MVPA's protective effects are most pronounced in women, younger adults, and those at socioeconomic extremes.

Sedentary behavior showed no independent association with CKM stage after adjustment for MVPA and covariates. In fully adjusted models (Table S9), neither 5–8 h/day (e.g., stage 1 OR 0.86, 95 % CI 0.63–1.18) nor ≥8 h/day (stage 2 OR 1.01, 95 % CI 0.71–1.44) differed from <5 h/day, and p for trend were consistently non-significant (all p for trend >0.23). Stratified models (Table S8) revealed that MVPA remained protective even among adults reporting ≥8 h/day of sitting—for instance, ≥150 min/week of MVPA was linked to lower odds of stage 1 (OR 0.47, 95 % CI 0.24–0.94), stage 2 (OR 0.37, 95 % CI 0.20–0.69), and stage 4 (OR 0.27, 95 % CI 0.08–0.96). Across parameterizations (categorical minutes/week and continuous MET-minutes/week), inferences were directionally concordant, supporting robustness to self-report specification. These findings indicate that prolonged sitting alone was not predictive of CKM once MVPA was accounted for, and that MVPA may mitigate risks among high-sedentary individuals.

## 4 Discussion

This study, utilizing a substantial, nationally representative sample of U.S. adults, revealed a negative correlation between time spent engaging in moderate-to-vigorous physical activity (MVPA) and the risk of advancing through different stages of CKM syndrome. Trend analysis demonstrated a relationship between MVPA volumes and CKM stage: the more time spent on MVPA, the less likely CKM was. The RCS curve shows that there is a linear relationship between MVPA and CKM stage (2–4), and a nonlinear relationship between MVPA and stage 4. Positive results from trend analysis were also observed in some subgroup analyses. These results underscore the significance of MVPA as a crucial protective factor against CKM.

Recent work has already examined the association between physical activity and early CKM syndrome. In particular, Wang et al. analyzed NHANES 2007–2018 and reported a nonlinear dose–response between MVPA (MET-min/week) and early CKM (stage 0–2) [9]. Beyond the early-stage analysis by Wang et al., prior literature has primarily described exercise benefits separately within cardiovascular or renal domains. For example, Beddhu et al. reported protective associations of physical activity in CKD and non-CKD populations [20], while Rangaswami et al. synthesized the bidirectional pathophysiology of cardiorenal syndrome, calling for more integrated preventive approaches [21]. By leveraging the 2023 AHA CKM staging applied to a nationally representative sample, our study is, to our knowledge, the first to quantify stage-specific associations of PA across the entire CKM continuum. In contrast, our study extends this literature by (i) applying the 2023 AHA CKM staging across all stage (0–4), including advanced stage (3–4); (ii) using a longer time window (NHANES 2007–2020); (iii) jointly modeling MVPA and SB in survey-weighted multinomial models; and (iv) providing stage-specific restricted cubic spline and subgroup analyses by age, sex, and socioeconomic status. These features clarify stage-dependent patterns and strengthen the translational relevance of our findings. While an increasing number of studies have indicated the effects of MVPA on positive metabolic, cardiovascular, and renal outcomes [22,23], limited research has examined CKM multimorbidity across various levels of severity. Consistent with extensive prior evidence summarized in our Introduction, higher MVPA is broadly linked to favorable cardiometabolic and renal profiles; here we focus on how these benefits map onto CKM stage [[24], [25], [26], [27], [28]]. A negative relationship exists between the level of physical activity and prevalence of metabolic syndrome (MetS) [2,24]. Our findings parallel prior work on MetS, where higher physical activity has consistently related to lower MetS risk in prospective analyses and reviews [[29], [30], [31]]. Distinct from MetS-only studies, our analysis uses the AHA 2023 CKM staging that integrates cardiovascular and kidney domains with metabolic risk, enabling stage-resolved estimates spanning subclinical (stage 3) to clinical CVD (stage 4). This broader, multimorbidity-centered framework likely explains the stronger gradients observed at later CKM stage [[30], [31], [32]]. Evidence from cardiorenal cohorts points in the same direction. In NHANES-based analyses, Beddhu et al. reported that greater habitual physical activity was associated with lower mortality in both CKD and non-CKD populations [20]. Consistent with the cardiorenal interplay outlined by the AHA scientific statement [33], we observed the steepest MVPA gradients at CKM stage 3 (subclinical CVD), supporting the notion that activity may be particularly impactful when cardiorenal vulnerability emerges, whereas our CKM staging demonstrates these benefits across a broader multimorbidity spectrum. MacKinnon HJ showed that the amount of physical activity can potentially be modified, with insufficient levels and reduced physical capabilities linked to higher mortality rates and negative clinical outcomes, including the deterioration of kidney function [6,34]. Our study further extended these findings by demonstrating that MVPA duration is predisposed to the coexistence of CKM. Beyond these statistical associations, potential biological mechanisms may underlie the observed links. From a biological standpoint, insufficient physical activity is associated with the accumulation of excess adipose tissue [35]. Dysfunctional adipose tissue emits inflammatory and oxidative compounds that damage the blood vessels, cardiac muscles, and renal organs [[36], [37], [38], [39]]. Excessive fat accumulation in various parts of the body can have detrimental effects on organ functions. When fat builds up around the heart, specifically in the epicardium and pericardium, it can cause serious cardiac issues, such as arrhythmias, reduced heart function, and coronary artery disease. Similarly, fat deposits in and around the kidneys can lead to hypertension and erratic blood pressure changes [[38], [39], [40]]. Numerous studies have indicated that obesity plays a central role in the development of CKM syndrome [41]. Physical activity, when used as a primary method to prevent obesity, can effectively enhance the regulation of hippocampal function, promote neurogenesis and synaptic plasticity, improve cerebral blood flow, reduce blood glucose and blood pressure levels, decrease cardiovascular risk, and diminish proinflammatory activity [42]. Our findings emphasize the significance of understanding MVPA as a preventive strategy for CKM.

We also observed that the amount and intensity of MVPA exerted differential effects across CKM stage. For those in earlier stages (1 and 2), greater weekly MVPA was associated with notably lower risk, whereas individuals at stages 3 and 4 appeared to benefit from any additional MVPA. According to the 2018 Physical Activity Guidelines issued by the US Department of Health and Human Services (HHS), adults aged 18 to 65 years should engage in at least 150 min of moderate-intensity exercise each week [43,44]. A study in the United States showed that contrary to current recommendations, exercise levels in the general population remain suboptimal and indicate that less than 50 % of adults reach the recommended threshold for physical activity. Furthermore, one to four young people have reported no recent engagement in physical exercise [26,27,45], and an inactive lifestyle is an alarming risk factor in the 21st century. Insufficient physical activity is a primary factor in the development of numerous chronic health issues, beyond osteoporosis, degenerative joint conditions, and diminished neuromuscular power. Lack of exercise also contributes to atherosclerosis, cardiovascular ailments, kidney problems, metabolic disorders, cognitive decline, and malignancies. The absence of regular physical activity has far-reaching consequences for overall health and well-being [42]. During brief periods of intense physical exertion, glucose and other carbohydrates serve as the primary energy sources for the body. In contrast, lipids become the main fuel during extended exercise sessions or when engaging in physical activities of low to moderate intensity [6]. Individuals who have undergone training exhibit a superior ability to utilize lipids as an energy source during exercise compared to their untrained counterparts. Additionally, these trained subjects demonstrated a more rapid return to baseline serum levels of lipid metabolites following physical activity [46]. Therefore, long-term regular exercise can prevent the accumulation of lipids and their metabolites in blood vessels and adipose tissue, thus reducing hypertension, obesity, insulin resistance, inflammation, and oxidative stress, and lowering the risk of heart disease, renal disease, metabolic disease and death [24]. The trend analysis results of our study corroborate this situation, indicating that participants who spent more time engaging in MVPA during the same stage had lower odds of experiencing CKM. Despite the crucial role of physical activity in influencing disease onset and progression, the current medical landscape predominantly emphasizes pharmacological approaches to treating illnesses, while inadequately addressing lifestyle interventions [47]. In the general population, Many people are overly reliant on medications to control weight, lower blood glucose, and improve cardiovascular function; however, medications have side effects that are unknown to us. However, substantial evidence suggests that PA is broadly as effective as pharmacological interventions in maintaining weight and preventing diabetes, cardiovascular disease, and associated premature mortality [48]. Therefore, understanding the relationship between the distinct phases of CKM syndrome and MVPA level is essential for risk assessment, early intervention, and mortality reduction.

Subgroup analyses showed that individuals with higher SB also experienced lower CKM risk when they engaged in any level of MVPA. The protective effect of MVPA was more pronounced among women and younger adults (<60 years). With respect to socioeconomic status, benefits were observed at both low and high PIR strata, though effect sizes varied by stage. These subgroup findings should be interpreted cautiously, as uneven data distribution could affect statistical accuracy. For instance, women's complex hormonal changes and differing exercise patterns, the smaller sample sizes of certain income groups, and the absence of stage 0 participants in older age strata may introduce bias. Nonetheless, these variations highlight the importance of tailoring interventions to demographic and socioeconomic contexts.

In SB main-effect models that additionally included MVPA (MET-min/week), neither 5–8 h/day nor ≥8 h/day of SB was associated with CKM stage 1–4 when compared with <5 h/day (e.g., stage 1: OR = 0.86 [0.63–1.18] and 0.98 [0.68–1.40], p for trend = 0.64; stage 2: 0.96 [0.71–1.31] and 1.01 [0.71–1.44], p for trend = 0.76; stage 3: 1.05 [0.43–2.53] and 1.24 [0.53–2.90], p for trend = 0.23; stage 4: 0.74 [0.41–1.35] and 0.85 [0.48–1.48], p for trend = 0.58; Table S9). In contrast, within each SB stratum, higher MVPA remained consistently protective—most notably among adults sitting ≥8 h/day, where ≥150 min/week of MVPA was associated with lower odds of CKM stage 1, 2, and 4 (stage 1: OR = 0.47 [0.24–0.94]; stage 2: 0.37 [0.20–0.69]; stage 4: 0.27 [0.08–0.96]; Table S8). These patterns indicate that (i) collinearity and behavioral overlap between MVPA and SB attenuate SB's independent signal when both are modeled; (ii) our single-item, self-reported SB measure likely introduced non-differential misclassification, biasing estimates toward the null; and (iii) higher MVPA may offset risks associated with prolonged sitting, highlighting that the 24-h movement composition (i.e., the joint MVPA–SB pattern) may matter more than SB in isolation. These null main effects after MVPA adjustment may appear to contrast with the AHA advisory summarizing positive associations between sedentary time and CVD incidence/mortality [33]. Differences in exposure assessment (single-item self-reported SB here vs. device-measured SB in many advisory-cited cohorts), outcomes (CKM staging vs. clinical events/mortality), and mutual adjustment for MVPA likely explain the discrepancy; notably, our stratified results indicate that higher MVPA mitigates risk even at ≥8 h/day of sitting [33].

Our stage-resolved findings support incorporating brief MVPA screening and counseling into CKM risk assessment. For adults with CKM stage 0–2, prioritizing attainment of ≥150 min/week of MVPA (or equivalent) may help prevent progression; for stages 3–4, any increase from inactivity appears beneficial, aligning with guidance to start low and progress gradually, with referral to supervised programs when needed [49]. Embedding MVPA prompts in electronic health records, offering step-based goals (e.g., 7000–8000 steps/day), and framing ‘sit-less, move-more’ as time reallocation (breaking up ≥8 h/day of sitting with light-to-moderate activity) are pragmatic strategies. Public-health messaging can target high-risk groups identified in our subgroups (e.g., older adults, lower PIR) to reduce disparities.

Key strengths include the novelty of applying the 2023 AHA CKM staging framework to NHANES 2007–2020, the large nationally representative sample (*n* = 18,405) with survey-weighted analyses, and clear stage-specific estimates (e.g., OR 0.09 for stage 3, Fig. 4) that align with biological mechanisms such as adipose tissue dysfunction.

Our study has several limitations. First, although NHANES is nationally representative of the U.S. civilian noninstitutionalized population, external validity to non-U.S. settings may be limited given cross-country differences in health-care systems, physical-activity norms, and CKM stage distributions. Second, the NHANES survey lacks continuous data on CKM stage progression and associated conditions and relies on self-reported information for some CKM indicators, potentially resulting in misclassification and recall bias. Additionally, the survey did not include data on certain cardiovascular diseases, such as atrial fibrillation and peripheral artery disease, which may lead to an underestimation of the prevalence of CKM stage 4. Third, Physical activity and sedentary behavior were self-reported (with a single-item measure for SB), which introduces non-differential misclassification likely biasing estimates toward the null—particularly for SB—and may partly explain attenuated SB main effects after MVPA adjustment. Differential misclassification across subgroups (e.g., by age, BMI, or education) is also possible; we therefore emphasize effect sizes with 95 % CIs, triangulate findings across MVPA parameterizations (categorical minutes/week and continuous MET-minutes/week with RCS), and interpret results cautiously. Fourth, the study had a substantial overall sample size; certain subgroups (such as elderly participants and the high PIR group with CKM stage 0) had relatively small numbers, potentially resulting in a sparse data bias. Fifth, because NHANES is cross-sectional, temporality cannot be established and reverse causation is plausible—for example, individuals with advanced CKM may reduce MVPA and increase sedentary time due to symptoms or treatment burden. Despite extensive adjustment, residual confounding cannot be excluded. Unmeasured or imprecisely measured factors—such as medication adherence or intensity, comorbidity severity, dietary quality, sleep and mental health, occupational activity, health-care access, or genetic predisposition—may influence both MVPA/SB and CKM. Accordingly, the observed associations should be interpreted as non-causal. Future longitudinal cohorts and quasi-experimental approaches (e.g., target-trial emulations) are needed to clarify the directionality and magnitude of these relationships. Despite offering valuable insights, our study has limitations that should be considered when evaluating the findings.

From an economic perspective, CKM-related diseases impose a major burden on healthcare systems. In the United States, the direct annual costs of cardiovascular disease exceed $200 billion [50]. Physical inactivity itself is estimated to cost billions globally each year [51], underscoring that promoting PA is not only clinically beneficial but also highly cost-effective. These stage-specific insights can inform brief screening and tailored counseling (e.g., targeting ≥150 min/week for stages 0–2 and ‘any-increase’ messaging for stages 3–4), thereby enhancing the clinical and public-health management of CKM.

## 5 Conclusion

In conclusion, our study found that physical activity was inversely associated with CKM risk, and that greater time in MVPA corresponded to lower odds across CKM stage.

The following are the supplementary data related to this article.

Table S1 Definitions of CKM indicators Table S1

Table S2 Detailed algorithm of the simplified 10-year CVD risk models Table S2

Table S3 Levels of MVPA level in relation to CKM stage 1 to 4 in unadjusted and partially adjusted models Table S3

Table S4 Metabolic equivalent (MET) minutes of MVPA in relation to CKM stage 1 to 4 in different models Table S4

Table S5 Levels of MVPA in relation to CKM stage 1–4 by sex subgroups in all-adjusted model Table S5

Table S6 Levels of MVPA in relation to CKM stage 1–4 by age subgroups in all-adjusted model Table S6

Table S7 Levels of MVPA in relation to CKM stage 1–4 by PIR subgroups in all-adjusted model Table S7

Table S8 Levels of SB level in relation to CKM stage 1 to 4 in fully adjusted model Table S8

Table S9 Levels of SB level in relation to CKM stage 1 to 4 in fully adjusted model Table S9

## CRediT authorship contribution statement

**Shuo Pang:** Writing – original draft, Data curation. **Yuxi Dongye:** Writing – original draft, Software. **Yingwei Bi:** Writing – review & editing, Resources, Data curation. **Jianbo Wang:** Writing – review & editing, Supervision, Project administration, Methodology, Funding acquisition.

## Author statement

This manuscript has not been published or being considered for publication elsewhere in whole or in part.

## Consent for publication

Not applicable.

## Ethical approval and consent to participate

Data analyzed in this study were obtained from NHANES. Protocols involved were approved by the National Center for Health Statistics (NCHS) Research Ethics Review Board (ERB), and consent from all participants was documented.

## Funding

This work was supported by grants from the 10.13039/501100001809National Natural Science Foundation of China (No. 82173121).

## Declaration of competing interest

All listed authors declare that they have no known competing financial interests or personal relationships that could have appeared to influence the work reported in this paper.

## Data Availability

The datasets generated during the current study are available in the NHANES repository: https://www.cdc.gov/nchs/nhanes/index.htm.
