# Supplementary material for: Association of physical activity and sedentary behavior with stages of cardiovascular–kidney–metabolic syndrome among U.S. adults: NHANES 2007–2020
Source: Am Heart J Plus. 2025 Oct 14;60:100639. doi: 10.1016/j.ahjo.2025.100639 (PMC12554204; doi:10.1016/j.ahjo.2025.100639)
Supplement: Table S1 — Definitions of CKM indicators [file mmc1.docx]

**Table S1 Definitions of CKM indicators**

| CKM conditions | CKM indicators | Definitions of CKM indicators |
| --- | --- | --- |
| CVD | Clinical CVD | History of chronic heart failure, coronary heart disease, heart attack, or stroke |
|  | Subclinical CVD | Any of the following criterion is met:  1) Very high-risk CKD in KDIGO classification: UACR ≥ 300 mg/g and eGFR ≤ 45-59 ml/min/1.73m^2^, UACR ≥ 30 mg/g and eGFR ≤ 30-44 ml/min/1.73m^2^, or eGFR ≤29 ml/min/1.73m^2^.  2) Predicted 10-year CVD risk ≥ 20% |
| Chronic kidney diseases  (CKD) | CKD | Moderate-to-high-risk CKD in KDIGO classification: UACR ≥ 30 mg/g and eGFR ≥ 60 ml/min/1.73m^2^, UACR < 300 mg/g and eGFR ≤ 45-59 ml/min/1.73m^2^, or UACR < 30 mg/g and eGFR ≤ 30-44 ml/min/1.73m^2^. |
| Metabolic disorders | Overweight/obesity | BMI ≥25 kg/m^2^ (or ≥23 kg/m^2^ if Asian ancestry) * |
|  | Abdominal obesity | Waist circumference ≥88/102 cm in female/male (or if Asian ancestry ≥80/90 cm in female/male) |
|  | Prediabetes | Fasting blood glucose ≥ 100-124 mg/dL or HbA1c ≥5.7%-6.4% and without self-reported diagnosis of diabetes, use of insulin, or oral hypoglycemic agents |
|  | Diabetes | Fasting blood glucose ≥ 125 mg/dL or HbA1c ≥6.5% or self-reported diagnosis of diabetes, use of insulin, or oral hypoglycemic agents |
|  | Hypertension | SBP ≥130 mmHg or DBP ≥80 mmHg or self-reported diagnosis of hypertension or use of antihypertensive medications |
|  | Hypertriglyceridemia | Triglycerides ≥ 135 mg/dL |
|  | MetS | MetS is defined by the presence of 3 or more of the following：   1. Waist circumference ≥88/102 cm in female/male (or if Asian ancestry ≥80/90 cm in female/male). 2. HDL cholesterol ≥50/40 mg/dL in female/male. 3. Triglycerides ≥150 mg/dL. 4. Elevated blood pressure (SBP ≥130 mmHg or DBP ≥80 mmHg and/or use of antihypertensive medications) 5. Fasting blood glucose ≥100 mg/dL |

**Abbreviations:** BMI: body mass index; CKD: chronic kidney disease; CKM: cardiovascular-kidney-metabolic; CVD: cardiovascular disease; DBP: diastolic blood pressure; eGFR: estimated glomerular filtration rate; HDL: high-density lipoprotein; KDIGO: The Kidney Disease: Improving Global Outcomes; MetS: metabolic syndrome; SBP: systolic blood pressure; UACR: urinary albumin to creatinine ratio.

***** Asian was not listed as a separate race/ethnicity until NAHNES 2011-2012, therefore the uniform threshold for BMI and waist circumference was used in all participants in NHANES 2007-2010.
