# Supplementary material for: Association of physical activity and sedentary behavior with stages of cardiovascular–kidney–metabolic syndrome among U.S. adults: NHANES 2007–2020
Source: Am Heart J Plus. 2025 Oct 14;60:100639. doi: 10.1016/j.ahjo.2025.100639 (PMC12554204; doi:10.1016/j.ahjo.2025.100639)
Supplement: Table S2 — Detailed algorithm of the simplified 10-year CVD risk models [file mmc2.docx]

**Table S2 Detailed algorithm of the simplified 10-year CVD risk models**

| **Men** | **log-Odds** = -3.031168 + 0.7688528 × (age – 55) /10 + 0.0736174 × (TC – HDL-C – 3.5) – 0.0954431 × (HDL-C 1.3) /0.3 – 0.4347345 × (min(SBP, 110) – 110) /20 + 0.3362658 × (max(SBP, 110) – 130) /20 + 0.7692857 × (if diabetes) + 0.4386871 × (if current smoker) + 0.5378979 × (min(eGFR, 60) – 60) / -15 + 0.0164827 × (max(eGFR, 60) 90) / -15 + 0.288879 × (if using anti-hypertensive medication) – 0.1337349 × (if using statin) – 0.0475924 × (if using antihypertensive medication) × (max(SBP, 110) – 130) /20 + 0.150273 × (if using statin) × (TC – HDL-C – 3.5) – 0.0517874 × (age – 55) /10 × (TC – HDL-C – 3.5) + 0.0191169 × (age 55) /10 × (HDL-C – 1.3) /0.3 – 0.1049477 × (age – 55) /10 × (max(SBP, 110) – 130) /20 – 0.2251948 × (age – 55) /10 × (if diabetes) – 0.0895067 × (age – 55) /10 × (if current smoker) 0.1543702 × (age – 55) /10 × (min(eGFR, 60) – 60) / -15  **Risk** = exp(log-Odds) / (1 + exp(log-Odds)) |
| --- | --- |
| **Women** | **log-Odds** = -3.307728 + 0.7939329 × (age – 55) /10 + 0.0305239 × (TC – HDL-C – 3.5) – 0.1606857 × (HDL-C 1.3) /0.3 – 0.2394003 × (min(SBP, 110) – 110) /20 + 0.360078 × (max(SBP, 110) – 130) /20 + 0.8667604 × (if diabetes) + 0.5360739 × (if current smoker) + 0.6045917 × (min(eGFR, 60) – 60) / -15 + 0.0433769 × (max(eGFR, 60) – 90) / -15 + 0.3151672 × (if using anti-hypertensive medication) 0.1477655 × (if using statin) – 0.0663612 × (if using antihypertensive medication) × (max(SBP, 110) – 130) /20 + 0.1197879 × (if using statin) × (TC – HDL-C – 3.5) 0.0819715 × (age – 55) /10 × (TC – HDL-C – 3.5) + 0.0306769 × (age – 55) /10 × (HDL-C – 1.3) /0.3 – 0.0946348 × (age – 55) /10 × (max(SBP, 110) – 130) /20 – 0.27057 × (age – 55) /10 × (if diabetes) – 0.078715 × (age – 55) /10 × (if current smoker) – 0.1637806 × (age – 55) /10 × (min(eGFR, 60) – 60) / -15  **Risk** = exp(log-Odds) / (1 + exp(log-Odds)) |

**Abbreviations:**

eGFR: estimated glomerular filtration rate; HDL: high-density lipoprotein cholesterol; SBP: systolic blood pressure; TC: total cholesterol.
