# Supplementary material for: Association of physical activity and sedentary behavior with stages of cardiovascular–kidney–metabolic syndrome among U.S. adults: NHANES 2007–2020
Source: Am Heart J Plus. 2025 Oct 14;60:100639. doi: 10.1016/j.ahjo.2025.100639 (PMC12554204; doi:10.1016/j.ahjo.2025.100639)
Supplement: Table S3 — Levels of MVPA level in relation to CKM stage 1 to 4 in unadjusted and partially adjusted models [file mmc3.docx]

**Table S3 Levels of MVPA level in relation to CKM stage 1 to 4 in unadjusted and partially adjusted models**

|  |  | **Stage 1** | | | | **Stage 2** | | | | **Stage 3** | | | | **Stage 4** | | | | |
| --- | --- | --- | --- | --- | --- | --- | --- | --- | --- | --- | --- | --- | --- | --- | --- | --- | --- | --- |
| **Group** | **Characteristic** | **OR**^1^ | **95% CI**^1^ | **p-value** | **p for trend** | **OR**^1^ | **95% CI**^1^ | **p-value** | **p for trend** | **OR**^1^ | **95% CI**^1^ | **p-value** | **p for trend** | **OR**^1^ | **95% CI**^1^ | **p-value** | **p for trend** | |
| **Model 1** | **MVPA group^†^ (minutes/week)** |  |  | **0.011** | **0.003** |  |  | **<0.001** | **<0.001** |  |  | **<0.001** | **<0.001** |  |  | **<0.001** | **<0.001** | |
|  | 0 | — | — |  |  | — | — |  |  | — | — |  |  | — | — |  |  | |
|  | 1-149 | 0.71 | 0.44, 1.13 |  |  | 0.61 | 0.40, 0.94 |  |  | 0.39 | 0.24, 0.64 |  |  | 0.35 | 0.23, 0.53 |  |  | |
|  | >=150 | 0.62 | 0.45, 0.85 |  |  | 0.36 | 0.26, 0.50 |  |  | 0.22 | 0.16, 0.31 |  |  | 0.17 | 0.12, 0.23 |  |  | |
| **Model 2** | **MVPA group^†^ (minutes/week)** |  |  | **0.021** | **0.008** |  |  | **<0.001** | **<0.001** |  |  | **<0.001** | **<0.001** |  |  | **<0.001** | **<0.001** | |
|  | 0 | — | — |  |  | — | — |  |  | — | — |  |  | — | — |  |  | |
|  | 1-149 | 0.61 | 0.38, 1.00 |  |  | 0.71 | 0.45, 1.14 |  |  | 0.10 | 0.02, 0.41 |  |  | 0.34 | 0.18, 0.65 |  |  | |
|  | >=150 | 0.64 | 0.46, 0.88 |  |  | 0.49 | 0.35, 0.70 |  |  | 0.05 | 0.02, 0.14 |  |  | 0.20 | 0.12, 0.33 |  |  | |
| ^1^OR = Odds Ratio, CI = Confidence Interval | | | | | | | | | | | | | | | | | |  |

**Abbreviations:** CI: confidence interval; CKM: cardiovascular-kidney-metabolic; OR: odds ratio; MVPA: moderate-to-vigorous physical activity.

Model 1 only included MET minutes of MVPA; Model 2 were partially adjusted for age, sex, race/ethnicity.

**† MVPA was constructed by the summed time inactivity (0 minutes/week), low level of activity (1-149 minutes/week), and recommended activity level ( ≥ 150 minutes/week).**
