# Supplementary material for: Association of physical activity and sedentary behavior with stages of cardiovascular–kidney–metabolic syndrome among U.S. adults: NHANES 2007–2020
Source: Am Heart J Plus. 2025 Oct 14;60:100639. doi: 10.1016/j.ahjo.2025.100639 (PMC12554204; doi:10.1016/j.ahjo.2025.100639)
Supplement: Table S4 — Metabolic equivalent (MET) minutes of MVPA in relation to CKM stage 1 to 4 in different models [file mmc4.docx]

**Table S4 Metabolic equivalent (MET) minutes of MVPA in relation to CKM stage 1 to 4 in different models**

|  | | **Stage 1** | | | **Stage 2** | | | **Stage 3** | | | **Stage 4** | | |
| --- | --- | --- | --- | --- | --- | --- | --- | --- | --- | --- | --- | --- | --- |
| **Group** | **Characteristic** | **OR** | **95% CI** | **p-value** | **OR** | **95% CI** | **p-value** | **OR** | **95% CI** | **p-value** | **OR** | **95% CI** | **p-value** |
| **Model 1** | **MET total**  **(MET-minutes/week)** | 1.000002 | 0.999981, 1.000024 | 0.819 | 0.999978 | 0.999962, 0.999993 | **0.005** | 0.999910 | 0.999886, 0.999933 | **<0.001** | 0.999941 | 0.999917, 0.999964 | **<0.001** |
| **Model 2** | **MET total**  **(MET-minutes/week)** | 1.000000 | 0.999974, 1.000025 | 0.979 | 0.999989 | 0.999969, 0.999998 | **0.023** | 0.999877 | 0.999811, 0.999944 | **<0.001** | 0.999975 | 0.999942, 0.999995 | **0.020** |
| **Model 3** | **MET total**  **(MET-minutes/week)** | 0.999983 | 0.999957, 1.000010 | 0.207 | 0.999974 | 0.999954, 0.999995 | **0.018** | 0.999851 | 0.999794, 0.999908 | **<0.001** | 0.999960 | 0.999941, 0.999996 | **0.036** |
| Abbreviations: CI = Confidence Interval, OR = Odds Ratio | | | | | | | | | | | | | |

**Abbreviations:** CI: confidence interval; CKM: cardiovascular-kidney-metabolic; OR: odds ratio; MET total:Metabolic equivalent (MET) minutes of MVPA; PIR: poverty income ratio.

Model 1 only included MET minutes of MVPA; Model 2 were partially adjusted for age, sex, race/ethnicity; Model 3 were adjusted for age, sex, race/ethnicity, Healthy Eating Index-2015, educational level (above high school, high school or equivalent, under high school), marital status (married/cohabiting, never married, widowed/divorced/separated), tobacco use (current, former, and never), alcohol use (heavy, mild, moderate, and never), PIR [high (>3.49), low ( ≤1.49), medium (>1.49, < 3.49)], sedentary behavior time, and was categorized into three groups (< 5h/day, 5-8h/day, and >= 8h/day).
