# Supplementary material for: Association of physical activity and sedentary behavior with stages of cardiovascular–kidney–metabolic syndrome among U.S. adults: NHANES 2007–2020
Source: Am Heart J Plus. 2025 Oct 14;60:100639. doi: 10.1016/j.ahjo.2025.100639 (PMC12554204; doi:10.1016/j.ahjo.2025.100639)
Supplement: Table S5 — Levels of MVPA in relation to CKM stage 1–4 by sex subgroups in all-adjusted model [file mmc5.docx]

**Table S5 Levels of MVPA in relation to CKM stage 1-4 by sex subgroups in all-adjusted model**

|  |  | **Stage 1** | | | | **Stage 2** | | | | **Stage 3** | | | | **Stage 4** | | | |
| --- | --- | --- | --- | --- | --- | --- | --- | --- | --- | --- | --- | --- | --- | --- | --- | --- | --- |
| **Group** | **Characteristic** | **OR**^1^ | **95% CI**^1^ | **p-value** | **p for trend** | **OR**^1^ | **95% CI**^1^ | **p-value** | **p for trend** | **OR**^1^ | **95% CI**^1^ | **p-value** | **p for trend** | **OR**^1^ | **95% CI**^1^ | **p-value** | **p for trend** |
| **Female** | **MET_total** | 1.00 | 1.00, 1.00 | 0.516 |  | 1.00 | 1.00, 1.00 | 0.710 |  | 1.00 | 1.00, 1.00 | **0.005** |  | 1.00 | 1.00, 1.00 | 0.186 |  |
|  | **MVPA.group^†^ (minutes/week)** |  |  |  | 0.052 |  |  |  | **0.006** |  |  |  | **<0.001** |  |  |  | **0.007** |
|  | 0 | — | — |  |  | — | — |  |  | — | — |  |  | — | — |  |  |
|  | 1-149 | 0.60 | 0.32, 1.13 | 0.114 |  | 0.71 | 0.39, 1.31 | 0.270 |  | 0.18 | 0.03, 1.05 | 0.057 |  | 0.42 | 0.16, 1.13 | 0.084 |  |
|  | >=150 | 0.61 | 0.37, 1.00 | **0.050** |  | 0.50 | 0.31, 0.81 | **0.005** |  | 0.07 | 0.02, 0.26 | **<0.001** |  | 0.36 | 0.16, 0.80 | **0.014** |  |
| **Male** | **MET_total** | 1.00 | 1.00, 1.00 | 0.321 |  | 1.00 | 1.00, 1.00 | 0.168 |  | 1.00 | 1.00, 1.00 | **<0.001** |  | 1.00 | 1.00, 1.00 | 0.077 |  |
|  | **MVPA.group^†^ (minutes/week)** |  |  |  | 0.983 |  |  |  | 0.470 |  |  |  | 0.061 |  |  |  | 0.067 |
|  | 0 | — | — |  |  | — | — |  |  | — | — |  |  | — | — |  |  |
|  | 1-149 | 3.22 | 1.42, 7.29 | **0.006** |  | 1.57 | 0.86, 2.87 | 0.141 |  | 0.33 | 0.04, 2.75 | 0.302 |  | 0.88 | 0.38, 2.04 | 0.768 |  |
|  | >=150 | 1.60 | 1.03, 2.48 | **0.037** |  | 1.10 | 0.72, 1.68 | 0.643 |  | 0.09 | 0.01, 0.63 | **0.016** |  | 0.64 | 0.30, 1.36 | 0.241 |  |
| ^1^OR = Odds Ratio, CI = Confidence Interval | | | | | | | | | | | | | | | | | |

**Abbreviations:** CI: confidence interval; CKM: cardiovascular-kidney-metabolic; OR: odds ratio; PIR: poverty income ratio; MVPA: moderate-to-vigorous physical activity.

Models were adjusted for age, race/ethnicity, Healthy Eating Index-2015, educational level (above high school, high school or equivalent, under high school), marital status (married/cohabiting, never married, widowed/divorced/separated), tobacco use (current, former, and never), alcohol use (heavy, mild, moderate, and never), PIR [high (>3.49), low ( ≤1.49), medium (>1.49, < 3.49)], sedentary behavior time, and was categorized into three groups (< 5h/day, 5-8h/day, and >= 8h/day).

† MVPA was constructed by the summed time inactivity (0 minutes/week), low level of activity (1-149 minutes/week), and recommended activity level ( ≥ 150 minutes/week).
