# Supplementary material for: Association of physical activity and sedentary behavior with stages of cardiovascular–kidney–metabolic syndrome among U.S. adults: NHANES 2007–2020
Source: Am Heart J Plus. 2025 Oct 14;60:100639. doi: 10.1016/j.ahjo.2025.100639 (PMC12554204; doi:10.1016/j.ahjo.2025.100639)
Supplement: Table S6 — Levels of MVPA in relation to CKM stage 1–4 by age subgroups in all-adjusted model [file mmc6.docx]

**Table S6 Levels of MVPA in relation to CKM stage 1-4 by age subgroups in all-adjusted model**

|  |  | **Stage 1** | | | | **Stage 2** | | | | **Stage 3** | | | | **Stage 4** | | | |
| --- | --- | --- | --- | --- | --- | --- | --- | --- | --- | --- | --- | --- | --- | --- | --- | --- | --- |
| **Group** | **Characteristic** | **OR**^1^ | **95% CI**^1^ | **p-value** | **p for trend** | **OR**^1^ | **95% CI**^1^ | **p-value** | **p for trend** | **OR**^1^ | **95% CI**^1^ | **p-value** | **p for trend** | **OR**^1^ | **95% CI**^1^ | **p-value** | **p for trend** |
| **<60 (years)** | **MET total** | 1.00 | 1.00, 1.00 | 0.257 |  | 1.00 | 1.00, 1.00 | **0.009** |  | 1.00 | 1.00, 1.00 | 0.081 |  | 1.00 | 1.00, 1.00 | 0.342 |  |
|  | **MVPA group^†^ (minutes/week)** |  |  |  | **0.009** |  |  |  | **<0.001** |  |  |  | **<0.001** |  |  |  | **<0.001** |
|  | 0 | — | — |  |  | — | — |  |  | — | — |  |  | — | — |  |  |
|  | 1-149 | 0.66 | 0.38, 1.12 | 0.119 |  | 0.72 | 0.44, 1.20 | 0.203 |  | 0.15 | 0.04, 0.58 | **0.007** |  | 0.40 | 0.21, 0.74 | **0.004** |  |
|  | >=150 | 0.60 | 0.41, 0.87 | **0.009** |  | 0.41 | 0.28, 0.60 | **<0.001** |  | 0.13 | 0.06, 0.27 | **<0.001** |  | 0.27 | 0.17, 0.43 | **<0.001** |  |
| **>=60 (years)** | **MET total** | 1.00 | 1.00, 1.00 | 0.537 |  | 1.00 | 1.00, 1.00 | 0.659 |  | 1.00 | 1.00, 1.00 | 0.981 |  | 1.00 | 1.00, 1.00 | 0.866 |  |
|  | **MVPA group^†^ (minutes/week)** |  |  |  | 0.830 |  |  |  | 0.624 |  |  |  | 0.891 |  |  |  | 0.976 |
|  | 0 | — | — |  |  | — | — |  |  | — | — |  |  | — | — |  |  |
|  | 1-149 | 0.93 | 0.53, 1.62 | 0.790 |  | 1.00 | 0.89, 1.14 | 0.944 |  | 1.01 | 0.78, 1.30 | 0.929 |  | 1.00 | 0.81, 1.23 | 0.974 |  |
|  | >=150 | 0.94 | 0.60, 1.49 | 0.800 |  | 0.97 | 0.87, 1.09 | 0.635 |  | 0.97 | 0.80, 1.19 | 0.788 |  | 1.00 | 0.83, 1.21 | 0.981 |  |
| ^1^OR = Odds Ratio, CI = Confidence Interval | | | | | | | | | | | | | | | | | |

**Abbreviations: CI:** confidence interval; CKM: cardiovascular-kidney-metabolic; OR: odds ratio; PIR: poverty income ratio; MVPA: moderate-to-vigorous physical activity.

Models were adjusted for sex, race/ethnicity, Healthy Eating Index-2015, educational level (above high school, high school or equivalent, under high school), marital status (married/cohabiting, never married, widowed/divorced/separated), tobacco use (current, former, and never), alcohol use (heavy, mild, moderate, and never), PIR [high (>3.49), low ( ≤1.49), medium (>1.49, < 3.49)], sedentary behavior time, and was categorized into three groups (< 5h/day, 5-8h/day, and >= 8h/day).

† MVPA was constructed by the summed time inactivity (0 minutes/week), low level of activity (1-149 minutes/week), and recommended activity level ( ≥ 150 minutes/week).
