# Supplementary material for: Association of physical activity and sedentary behavior with stages of cardiovascular–kidney–metabolic syndrome among U.S. adults: NHANES 2007–2020
Source: Am Heart J Plus. 2025 Oct 14;60:100639. doi: 10.1016/j.ahjo.2025.100639 (PMC12554204; doi:10.1016/j.ahjo.2025.100639)
Supplement: Table S7 — Levels of MVPA in relation to CKM stage 1–4 by PIR subgroups in all-adjusted model [file mmc7.docx]

**Table S7 Levels of MVPA in relation to CKM stage 1-4 by PIR subgroups in all-adjusted model**

|  |  | **Stage 1** | | | | **Stage 2** | | | | **Stage 3** | | | | **Stage 4** | | | |
| --- | --- | --- | --- | --- | --- | --- | --- | --- | --- | --- | --- | --- | --- | --- | --- | --- | --- |
| **Group** | **Characteristic** | **OR**^1^ | **95% CI**^1^ | **p-value** | **p for trend** | **OR**^1^ | **95% CI**^1^ | **p-value** | **p for trend** | **OR**^1^ | **95% CI**^1^ | **p-value** | **p for trend** | **OR**^1^ | **95% CI**^1^ | **p-value** | **p for trend** |
| **High**  **(>3.49)** | **MET total** | 1.00 | 1.00, 1.00 | **0.049** |  | 1.00 | 1.00, 1.00 | 0.388 |  | 1.00 | 1.00, 1.00 | **0.003** |  | 1.00 | 1.00, 1.00 | 0.789 |  |
|  | **MVPA group^†^ (minutes/week)** |  |  |  | 0.097 |  |  |  | **0.020** |  |  |  | **0.002** |  |  |  | **0.047** |
|  | 0 | — | — |  |  | — | — |  |  | — | — |  |  | — | — |  |  |
|  | 1-149 | 0.54 | 0.24, 1.22 | 0.138 |  | 0.68 | 0.30, 1.51 | 0.335 |  | 0.01 | 0.00, 0.72 | **0.035** |  | 0.15 | 0.04, 0.63 | **0.011** |  |
|  | >=150 | 0.54 | 0.28, 1.04 | 0.067 |  | 0.45 | 0.24, 0.87 | **0.017** |  | 0.01 | 0.00, 0.12 | **<0.001** |  | 0.27 | 0.06, 1.27 | 0.095 |  |
| **Medium**  **(>1.39,<=3.49)** | **MET total** | 1.00 | 1.00, 1.00 | 0.813 |  | 1.00 | 1.00, 1.00 | 0.417 |  | 1.00 | 1.00, 1.00 | 0.105 |  | 1.00 | 1.00, 1.00 | 0.212 |  |
|  | **MVPA group^†^ (minutes/week)** |  |  |  | 0.505 |  |  |  | 0.141 |  |  |  | **0.036** |  |  |  | 0.093 |
|  | 0 | — | — |  |  | — | — |  |  | — | — |  |  | — | — |  |  |
|  | 1-149 | 0.64 | 0.24, 1.69 | 0.362 |  | 0.73 | 0.33, 1.64 | 0.446 |  | 0.44 | 0.06, 3.06 | 0.402 |  | 0.83 | 0.16, 4.21 | 0.815 |  |
|  | >=150 | 0.77 | 0.39, 1.51 | 0.442 |  | 0.63 | 0.33, 1.23 | 0.174 |  | 0.18 | 0.03, 1.05 | 0.056 |  | 0.36 | 0.11, 1.16 | 0.087 |  |
| **Low**  **(≤1.39)** | **MET total** | 1.00 | 1.00, 1.00 | 0.947 |  | 1.00 | 1.00, 1.00 | 0.849 |  | 1.00 | 1.00, 1.00 | **0.031** |  | 1.00 | 1.00, 1.00 | 0.740 |  |
|  | **MVPA.group^†^ (minutes/week)** |  |  |  | 0.672 |  |  |  | 0.486 |  |  |  | **0.030** |  |  |  | 0.095 |
|  | 0 | — | — |  |  | — | — |  |  | — | — |  |  | — | — |  |  |
|  | 1-149 | 0.76 | 0.28, 2.04 | 0.574 |  | 1.13 | 0.45, 2.87 | 0.795 |  | 0.08 | 0.01, 0.75 | **0.028** |  | 0.70 | 0.21, 2.29 | 0.547 |  |
|  | >=150 | 0.88 | 0.46, 1.66 | 0.682 |  | 0.85 | 0.44, 1.65 | 0.628 |  | 0.13 | 0.02, 1.05 | 0.055 |  | 0.46 | 0.22, 0.95 | **0.037** |  |
| ^1^OR = Odds Ratio, CI = Confidence Interval | | | | | | | | | | | | | | | | | |

**Abbreviations:** CI: confidence interval; CKM: cardiovascular-kidney-metabolic; OR: odds ratio; PIR: poverty income ratio; MVPA: moderate-to-vigorous physical activity.

Models were adjusted for age, sex, race/ethnicity, Healthy Eating Index-2015, educational level (above high school, high school or equivalent, under high school), marital status (married/cohabiting, never married, widowed/divorced/separated), tobacco use (current, former, and never), alcohol use (heavy, mild, moderate, and never), sedentary behavior time, and was categorized into three groups (< 5h/day, 5-8h/day, and >= 8h/day).

† MVPA was constructed by the summed time inactivity (0 minutes/week), low level of activity (1-149 minutes/week), and recommended activity level ( ≥ 150 minutes/week).
