# Supplementary material for: Association of physical activity and sedentary behavior with stages of cardiovascular–kidney–metabolic syndrome among U.S. adults: NHANES 2007–2020
Source: Am Heart J Plus. 2025 Oct 14;60:100639. doi: 10.1016/j.ahjo.2025.100639 (PMC12554204; doi:10.1016/j.ahjo.2025.100639)
Supplement: Table S8 — Levels of SB level in relation to CKM stage 1 to 4 in fully adjusted model [file mmc8.docx]

**Table S8 Levels of SB level in relation to CKM stage 1 to 4 in fully adjusted model**

|  | | **Stage 1** | | | **Stage 2** | | | **Stage 3** | | | **Stage 4** | | |
| --- | --- | --- | --- | --- | --- | --- | --- | --- | --- | --- | --- | --- | --- |
| **Group** | **Characteristic** | **OR** | **95% CI** | **p-value** | **OR** | **95% CI** | **p-value** | **OR** | **95% CI** | **p-value** | **OR** | **95% CI** | **p-value** |
| **< 5h/day** | **MET total** | 1.00 | 1.00, 1.00 | 0.526 | 1.00 | 1.00, 1.00 | 0.546 | 1.00 | 1.00, 1.00 | **<0.001** | 1.00 | 1.00, 1.00 | 0.816 |
|  | **MVPA group (minutes/week)** |  |  |  |  |  |  |  |  |  |  |  |  |
|  | 0 | — | — |  | — | — |  | — | — |  | — | — |  |
|  | 1-149 | 0.54 | 0.17, 1.66 | 0.274 | 0.69 | 0.24, 1.99 | 0.485 | 0.06 | 0.00, 1.23 | 0.067 | 0.24 | 0.05, 1.11 | 0.067 |
|  | >=150 | 0.73 | 0.29, 1.82 | 0.493 | 0.64 | 0.28, 1.47 | 0.291 | 0.12 | 0.01, 1.09 | 0.059 | 0.30 | 0.09, 0.96 | **0.043** |
| **5-8h/day** | **MET total** | 1.00 | 1.00, 1.00 | 0.457 | 1.00 | 1.00, 1.00 | 0.076 | 1.00 | 1.00, 1.00 | **0.043** | 1.00 | 1.00, 1.00 | 0.375 |
|  | **MVPA group (minutes/week)** |  |  |  |  |  |  |  |  |  |  |  |  |
|  | 0 | — | — |  | — | — |  | — | — |  | — | — |  |
|  | 1-149 | 1.19 | 0.54, 2.62 | 0.661 | 1.33 | 0.60, 2.95 | 0.483 | 0.18 | 0.00, 10.2 | 0.400 | 0.55 | 0.10, 2.87 | 0.469 |
|  | >=150 | 1.22 | 0.59, 2.53 | 0.580 | 0.99 | 0.46, 2.13 | 0.985 | 0.01 | 0.00, 1.55 | 0.074 | 0.33 | 0.10, 1.15 | 0.081 |
| **>= 8h/day** | **MET total** | 1.00 | 1.00, 1.00 | 0.937 | 1.00 | 1.00, 1.00 | 0.776 | 1.00 | 1.00, 1.00 | 0.102 | 1.00 | 1.00, 1.00 | 0.185 |
|  | **MVPA group (minutes/week)** |  |  |  |  |  |  |  |  |  |  |  |  |
|  | 0 | — | — |  | — | — |  | — | — |  | — | — |  |
|  | 1-149 | 0.40 | 0.19, 0.81 | **0.013** | 0.59 | 0.30, 1.15 | 0.119 | 0.25 | 0.03, 2.08 | 0.197 | 0.30 | 0.11, 0.83 | **0.020** |
|  | >=150 | 0.47 | 0.24, 0.94 | **0.032** | 0.37 | 0.20, 0.69 | **0.002** | 0.14 | 0.02, 1.27 | 0.079 | 0.27 | 0.08, 0.96 | **0.043** |
| Abbreviations: CI = Confidence Interval, OR = Odds Ratio | | | | | | | | | | | | | |

**Abbreviations:** CI: confidence interval; CKM: cardiovascular-kidney-metabolic; OR: odds ratio; PIR: poverty income ratio; MVPA: moderate-to-vigorous physical activity.

Models were adjusted for age, sex, race/ethnicity, Healthy Eating Index-2015, educational level (above high school, high school or equivalent, under high school), marital status (married/cohabiting, never married, widowed/divorced/separated), tobacco use (current, former, and never), alcohol use (heavy, mild, moderate, and never), PIR [high (>3.49), low ( ≤1.49), medium (>1.49, < 3.49)].

† MVPA was constructed by the summed time inactivity (0 minutes/week), low level of activity (1-149 minutes/week), and recommended activity level ( ≥ 150 minutes/week).
