# Supplementary material for: Association of physical activity and sedentary behavior with stages of cardiovascular–kidney–metabolic syndrome among U.S. adults: NHANES 2007–2020
Source: Am Heart J Plus. 2025 Oct 14;60:100639. doi: 10.1016/j.ahjo.2025.100639 (PMC12554204; doi:10.1016/j.ahjo.2025.100639)
Supplement: Table S9 — Levels of SB level in relation to CKM stage 1 to 4 in fully adjusted model [file mmc9.docx]

**Table S9 Levels of SB level in relation to CKM stage 1 to 4 in fully adjusted model**

|  | **Stage 1** | | | | **Stage 2** | | | | **Stage 3** | | | | **Stage 4** | | | |
| --- | --- | --- | --- | --- | --- | --- | --- | --- | --- | --- | --- | --- | --- | --- | --- | --- |
| **Characteristic** | **OR** | **95% CI** | **p-value** | **p for trend** | **OR** | **95% CI** | **p-value** | **p for trend** | **OR** | **95% CI** | **p-value** | **p for trend** | **OR** | **95% CI** | **p-value** | **p for trend** |
| **SB^†^**  **(hours/day)** | 1.00 | 1.00, 1.00 | 0.195 |  | 1.00 | 1.00, 1.00 | 0.956 |  | 1.00 | 1.00, 1.00 | **0.041** |  | 1.00 | 1.00, 1.00 | 0.314 |  |
| **SB^†^ (hours/day)** |  |  |  | 0.635 |  |  |  | 0.764 |  |  |  | 0.233 |  |  |  | 0.575 |
| < 5h/day | — | — |  |  | — | — |  |  | — | — |  |  | — | — |  |  |
| 5-8h/day | 0.86 | 0.63, 1.18 | 0.346 |  | 0.96 | 0.71, 1.31 | 0.806 |  | 1.05 | 0.43, 2.53 | 0.920 |  | 0.74 | 0.41, 1.35 | 0.322 |  |
| >= 8h/day | 0.98 | 0.68, 1.40 | 0.900 |  | 1.01 | 0.71, 1.44 | 0.960 |  | 1.24 | 0.53, 2.90 | 0.608 |  | 0.85 | 0.48, 1.48 | 0.554 |  |
| Abbreviations: CI = Confidence Interval, OR = Odds Ratio | | | | | | | | | | | | | | | | |

**Abbreviations:** CI: confidence interval; CKM: cardiovascular-kidney-metabolic; OR: odds ratio; PIR: poverty income ratio; SB: sedentary behavior.

Models were adjusted for age, sex, race/ethnicity, Healthy Eating Index-2015, educational level (above high school, high school or equivalent, under high school), marital status (married/cohabiting, never married, widowed/divorced/separated), tobacco use (current, former, and never), alcohol use (heavy, mild, moderate, and never), PIR [high (>3.49), low ( ≤1.49), medium (>1.49, < 3.49)], MET total: Metabolic equivalent (MET) minutes of MVPA (moderate-to-vigorous physical activity)

† SB was constructed by the summed time inactivity < 5h/day, 5-8h/day, and >= 8h/day.
